# Coronary Artery Diffuse Aneurysmal Dilation in an Acute Myocardial Infarction Patient

**DOI:** 10.7759/cureus.4747

**Published:** 2019-05-24

**Authors:** Sherif Elhosseiny, Emad Barsoum, Ruben Kandov, James C Lafferty, Mohammad Zgheib

**Affiliations:** 1 Internal Medicine, Staten Island University Hospital, Staten Island, USA; 2 Cardiology, Staten Island University Hospital, Staten Island, USA; 3 Interventional Cardiology, Staten Island University Hospital, Staten Island, USA

**Keywords:** acute coronary syndrome, coronary artery aneurysm, atherosclerotic heart disease

## Abstract

Coronary artery aneurysm (CAA) is a rare disease that is associated with dangerous dormant complications. It is associated with atherosclerotic heart disease in half of the cases during a coronary angiogram. Currently, there are no guidelines for the management of such cases. We present a case of acute ST-segment elevation myocardial infarction in a male patient who was found to have diffuse aneurysmal dilation of the coronary arteries along with 100% occlusion of the right coronary artery. The complexity of the lesions caused him not to be a candidate for either percutaneous or surgical intervention. This raises an important question regarding treatment options in such a rare case.

## Introduction

Coronary artery aneurysm (CAA) is an uncommon anomaly defined as a segment of the coronary artery being more than 1.5 times the normal adjacent segment [1]. CAA is associated with coronary artery atherosclerosis that can result in hazardous outcomes. We report the case of a male patient who presented with an ST-segment elevation myocardial infarction who was found to have diffuse aneurysmal dilation of the coronary arteries along with a 100% occlusion of the right coronary artery. The unusual complexity of our case raises important questions regarding the patient’s treatment options.

## Case presentation

**Figure 1:**
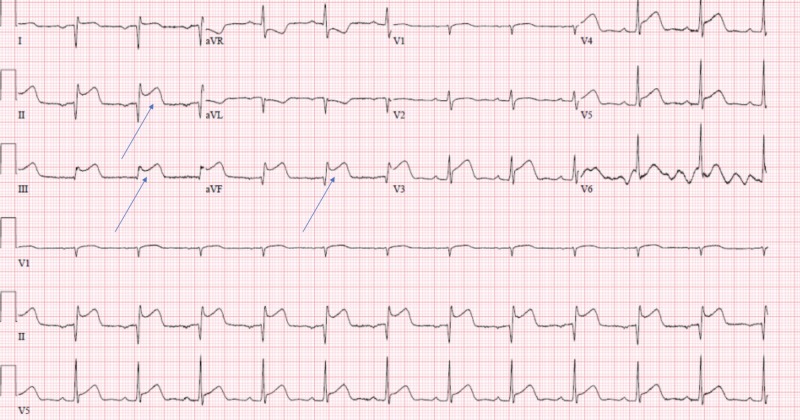
Electrocardiogram showing ST-segment elevation in leads II, III, and aVF

**Figure 2:**
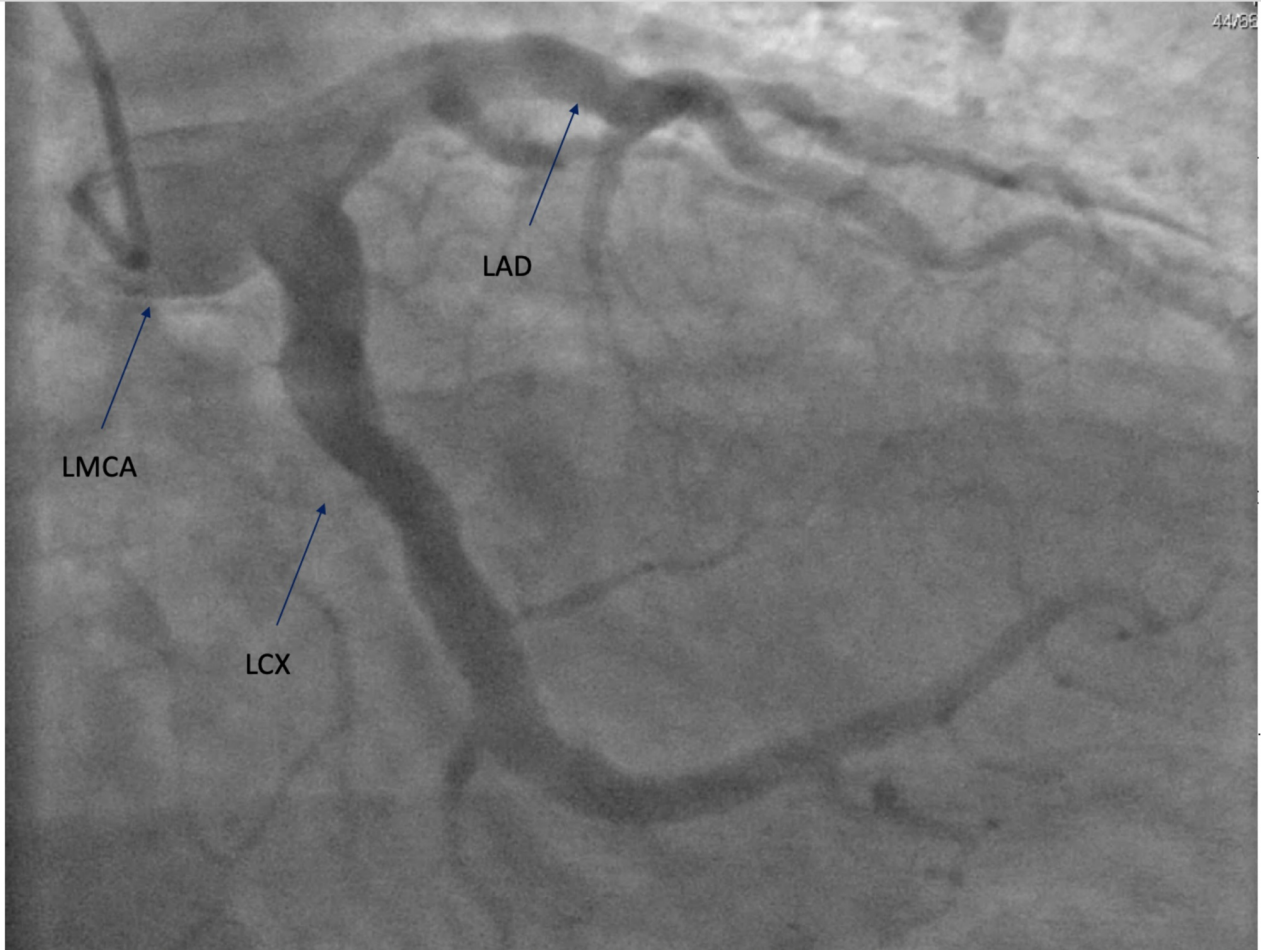
LMCA showing large aneurysmal dilation. LAD and LCX coronary arteries showing diffuse aneurysmal dilation affecting the entire length of the vessels. LMCA: left main coronary artery; LAD: left anterior descending; LCX: left circumflex

**Figure 3:**
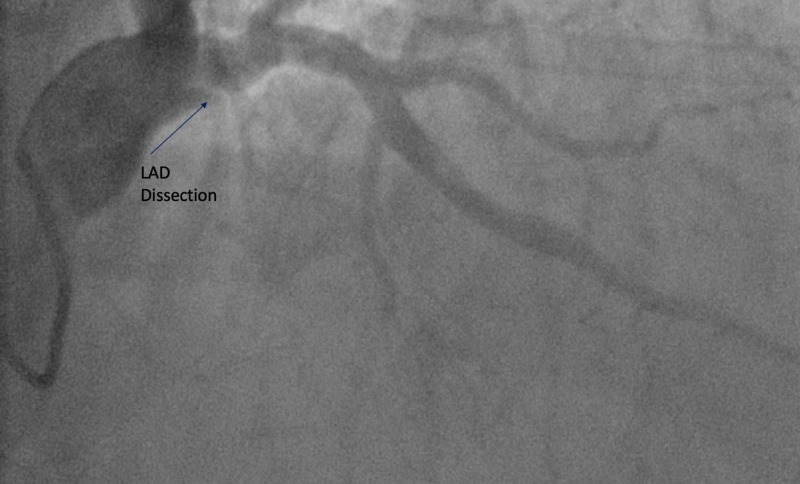
Proximal LAD coronary artery showing dissection LAD: left anterior descending

**Figure 4:**
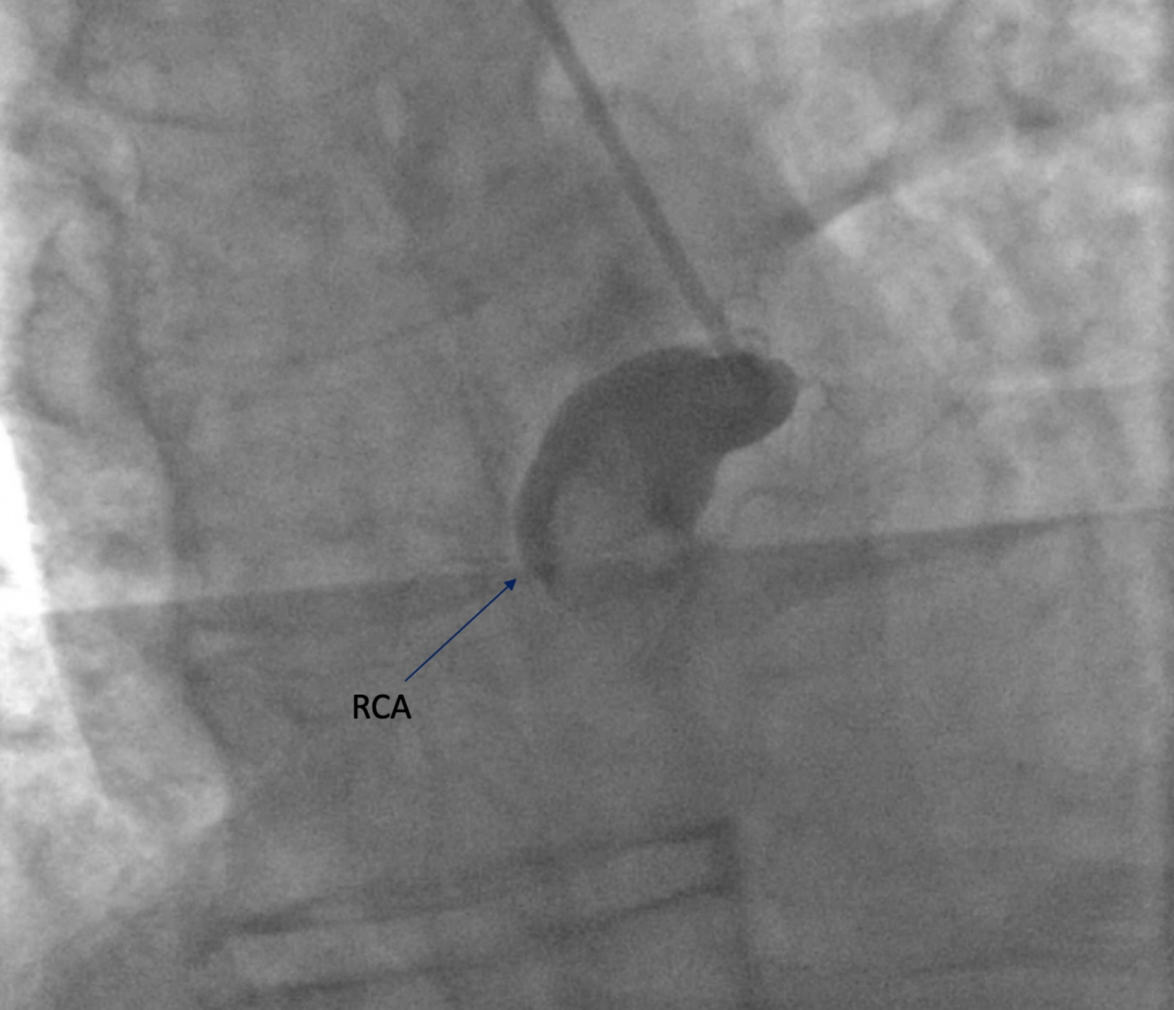
RCA showing proximal aneurysmal dilation with 100% occlusion with large thrombus RCA: right coronary artery

A 66-year-old male, former smoker, with a past medical history of hypertension, obesity, and obstructive sleep apnea presented with chest pain of three hours duration prior to presentation. Electrocardiogram (EKG) revealed an ST-segment elevation myocardial infarction in the inferior leads (Figure 1). The patient was rushed to cardiac catheterization for an angiogram. The angiogram has revealed large aneurysmal dilation of the left main coronary artery (LMCA) (Figure 2). The left anterior descending (LAD) and left circumflex (LCX) coronary arteries have shown diffuse aneurysmal dilation affecting the entire length of the vessels (Figure 2). There was rupture of plaque and dissection in the proximal LAD (Figure 3). The right coronary artery (RCA) has shown proximal aneurysmal dilation with 100% occlusion and with large thrombus that was the culprit lesion (Figure 4). The lesion was not amenable for intervention, as its diameter was larger than the available cardiac stents. Moreover, due to the complexity of the patient's lesion, he was not a candidate for surgical intervention. An intra-aortic balloon pump (IABP) was inserted to maintain the patient’s blood pressure and he was started on medical management with heparin infusion, dual antiplatelet therapy (DAPT), beta-blocker (BB), and high-intensity statin. An echocardiogram was done to reveal moderate left ventricular systolic dysfunction with an ejection fraction of 35%. Although there are no guidelines for the management of such a rare case, heparin was switched to rivaroxaban for long-term anticoagulation and IABP was removed. The patient was stable during the hospital stay and was discharged to follow-up as an outpatient.

## Discussion

Coronary artery aneurysm (CAA) is not a common pathology but a potentially serious disease. It was first described by Morgagni in 1761 [2]. CAA is defined as a segment of the coronary artery with a diameter more than 1.5 times the adjacent normal segment. The term ectasia is used when there is diffuse dilation that involves at least 50% of the vessel length. Its prevalence varies between studies and falls between 1.2% and 7.4% [3-5]. The pathology doesn’t affect the coronary arteries equally. The most frequently involved artery is the right coronary artery followed by the left anterior descending coronary artery and the left circumflex artery [1].

CAA can occur with no obvious background disease in a small percentage of the cases. However, CAA is associated with atherosclerotic heart disease in half of the case during an angiogram. It is presumed that CAA is a form of coronary artery disease [1, 4,6-7]. CAA is also associated with vasculitis (Kawasaki disease, Takayasu’s arteritis, and polyarteritis nodosa), systemic lupus erythematosus, Marfan syndrome, Ehlers-Danlos syndrome, trauma, and some infections [7-8]. The exact pathology is unknown, however, some possible mechanisms like genetic predisposition and arterial wall damage have been suggested [7].

The literature on CAA is limited to case reports and review articles. Patients who have CAA associated with atherosclerosis usually present with angina and myocardial infarction. Also, life-threatening complications such as rupture, vasospasm, and thromboembolism can occur [7]. An angiogram of the coronary arteries is the gold standard to diagnose CAA [6]; however, multi-slice computed tomography (MSCT) coronary angiogram can be an alternative diagnostic method [9]. There are a limited number of case reports about CAA that are associated with acute ST-segment elevation myocardial infarction [10-14].

Our case is unique, given the complexity of the anomalies and lesions presented by the angiogram. Currently, there is limited data regarding the treatment of CAA that is associated with acute myocardial infarction (MI). Other case reports have described cases of CAA with MI but were lacking the complexity of this case. Our patient had a left main coronary artery (LMCA) and left anterior descending (LAD) and left circumflex (LCX) coronary arteries diffuse aneurysmal dilation affecting the entire length of the vessels with rupture of plaque in the proximal LAD. The right coronary artery (RCA) has shown proximal aneurysmal dilation with 100% occlusion and a large thrombus that wasn’t amenable to either percutaneous or surgical intervention. The last resort was to start him on anticoagulation with a heparin drip followed by rivaroxaban upon discharge. These kinds of CAA cases associated with acute MI raise important questions regarding management options.

## Conclusions

Coronary artery aneurysm is a rare disease found on a small percentage of coronary angiograms. It is associated with coronary artery disease in half of the cases during an angiogram. Acute myocardial infarction in a large coronary aneurysm is a very rare condition. Medical management is the last resort and the role of anticoagulation remains controversial. Our case is of particular interest, as there are no available guidelines for management. Dual antiplatelet therapy (DAPT), anticoagulation, and continuous follow-ups are the available options for such a rare case.
